# rMSIfragment: improving MALDI-MSI lipidomics through automated in-source fragment annotation

**DOI:** 10.1186/s13321-023-00756-2

**Published:** 2023-09-15

**Authors:** Gerard Baquer, Lluc Sementé, Pere Ràfols, Lucía Martín-Saiz, Christoph Bookmeyer, José A. Fernández, Xavier Correig, María García-Altares

**Affiliations:** 1 Department of Electronic Engineering, University Rovira I Virgili, Tarragona, Spain; 2 Spanish Biomedical Research Center in Diabetes and Associated Metabolic Disorders (CIBERDEM), Madrid, Spain; 3 Institut D’Investigacio Sanitaria Pere Virgili, Tarragona, Spain; 4 Department of Physical Chemistry, Faculty of Science and Technology, University of the Basque Country (UPV/EHU), Leioa, Spain; 5 Institute of Hygiene, University of Münster, Münster, Germany

**Keywords:** In-source fragmentation, In-source decay, Lipids, Annotation, Mass spectrometry imaging, MALDI, Computation, Cheminformatics, Bioinformatics

## Abstract

Matrix-Assisted Laser Desorption Ionization Mass Spectrometry Imaging (MALDI-MSI) spatially resolves the chemical composition of tissues. Lipids are of particular interest, as they influence important biological processes in health and disease. However, the identification of lipids in MALDI-MSI remains a challenge due to the lack of chromatographic separation or untargeted tandem mass spectrometry. Recent studies have proposed the use of MALDI in-source fragmentation to infer structural information and aid identification. Here we present rMSIfragment, an open-source R package that exploits known adducts and fragmentation pathways to confidently annotate lipids in MALDI-MSI. The annotations are ranked using a novel score that demonstrates an area under the curve of 0.7 in ROC analyses using HPLC–MS and Target-Decoy validations. rMSIfragment applies to multiple MALDI-MSI sample types and experimental setups. Finally, we demonstrate that overlooking in-source fragments increases the number of incorrect annotations. Annotation workflows should consider in-source fragmentation tools such as rMSIfragment to increase annotation confidence and reduce the number of false positives.

**Supplementary Information:**

The online version contains supplementary material available at 10.1186/s13321-023-00756-2.

## Introduction

Matrix-Assisted Laser Desorption Ionization Mass Spectrometry Imaging (MALDI-MSI) is an analytical technique used in biochemical and clinical studies to reveal the chemical composition and spatial information of organic tissues [7, 25, 28, 38, 53]. It provides valuable information in many applications, including the understanding and diagnosis of complex diseases such as cancer [10, 13, 15, 29, 32, 35, 39], diabetes [6, 22, 33, 57], Alzheimer’s [27, 30, 31] and infectious diseases [36, 54]. In particular, the study of lipids is pivotal, as they play important roles in different pathways in health and disease [34].

Despite the surge of MALDI-MSI’s popularity, associating each mass-to-charge (*m/z*) signal with univocal molecular identifications remains challenging: (1) samples include thousands of molecules; (2) each molecule is responsible for several MS signals (e.g. isotopes, adducts and, in-source fragments); and (3) isomers and isobars cannot be resolved using only MS1 [8].

Traditional mass spectrometry techniques rely on chromatographic separation (LC–MS, GC–MS) for sample simplification [63]. However, MALDI-MSI does not include such separation steps. Alternatively, tandem mass spectrometry can augment the depth of the chemical analysis by providing fragmentation information on single molecules. Many MALDI-MSI instruments are equipped with tandem-MS capabilities (Bruker’s ultrafleXtreme, Thermo Scientific’s MALDI LTQ Orbitrap XL, or Waters’ MALDI SYNAPT G2-Si) but untargeted imaging MS/MS is not routinely feasible due to (1) prohibitive running times, (2) limited parental ion selectivity and intensity, and (3) increased data size and complexity. For all these reasons, untargeted fragmentation of all ions in a sample is only possible using highly specialized instrumental setups [18, 24].

Traditionally, In-Source Decay (ISD) or In-Source Fragmentation (ISF) (i.e. the natural and unavoidable generation of fragments inside the MALDI ion source) has been considered an undesired artifact and thus minimized [26]. ISD depends mainly on the chemical structure of the analyte and ionization conditions such as ionization temperature or voltage [26]. It can be problematic in the study of lipids, as several fragmentation pathways lead to isobaric lipid species. As an example, phosphatidic acid (PA) fragments can be produced in-source from their phosphatidylserine (PS) counterparts, and phosphatidylcholine (PC) in-source fragments are isobaric to endogenous phosphatidylethanolamine (PE) species [26]. These known lipid fragmentation pathways result in falsely low concentrations of lipids suffering from ISD and falsely high concentrations of lipids overlapping with isobaric in-source fragments. Additionally, if not properly annotated and removed, in-source fragments can yield an increased number of incorrect annotations using common MALDI-MSI annotation tools such as LipoStar, METASPACE, and rMSIannotation [42, 49, 55].

Nevertheless, recent studies have advocated for the use of well-characterized MALDI-ISD as a fast way of obtaining complementary fragmentation information to assist identification in the analysis of large molecules [5, 12, 37] and even lipids [58]. Some of these studies use automated protein ISD annotation tools like ProteinProspector (Baker, P.R. and Clauser, K.R. http://prospector.ucsf.edu) or DataAnalysis (Bruker Daltonics) but heavily rely on manual annotation. The use of ISD to strengthen identification has also been applied in MALDI-MSI in the field of top-down proteomics. Debois et al. demonstrated the use of ISD for in-situ de novo sequencing of several proteins on a porcine eye lens and a mouse brain slice [14, 64]. Similarly, Ait-Belkacem et al. [2] used ISD to identify several tumorigenic proteins in glioblastoma mouse brain tissue with MALDI-MSI. Franceschi et al. proposed a semi-automated workflow for in-source fragmentation annotation based on initial manual annotation of parental metabolites followed by an Intensity Correlation Analysis to find ions with a high spatial correlation and thus assumed to be in-source fragments. They used this approach to image flavonols and dihydrochalcones in golden apple samples [19].

Recently, Garate et al. [20], reported the MALDI-MSI in-source fragmentation pathways and adduct formation of the 17 main lipid classes (Additional file 1: Figure S1 and Table S1-S2). Here we propose rMSIfragment, a software solution that exploits these known in-source fragmentation pathways to increase confidence in lipid annotations. Our novel ranking score combines the times a given lipid has been found in the dataset (adducts and in-source fragments) and their spatial correlation to filter out unlikely lipids. After validation with HPLC and 2 different Target-Decoy approaches, rMSIfragment demonstrates an Area Under the Curve (AUC) of over 0.7 on multiple sample types and experimental conditions. We also find that ISD-agnostic annotation tools like METASPACE can falsely annotate in-source fragments as endogenous lipids.

## Algorithm description

### Input and output format

As input, the user should provide the ppm tolerance for exact mass searches against the database and an MSI dataset in the rMSIproc [45] peak matrix format ([# pixels] x [# *m/z*] intensity matrix). Refer to the public repository (https://github.com/prafols/rMSIproc) for instructions on how to convert profile and centroid mode.imZML files to the peak matrix format. The theoretical fragmentation pathways and adducts for each lipid class [20] (Additional file 1: Figure S1 and Table S1-S2) are already included in rMSIfragment.

The algorithm produces a table of annotations (lipid annotation, number of carbons, number of double bonds, and molecular formula) for each of the monoisotopic masses found in the data set. Each annotation is associated with a likelihood score to assist manual curation. The resulting table can be exported as a.csv file (Additional file 1: Table S3). Additionally, the annotation results can be used to generate a molecular network graph.

### Database search and likelihood score

**Fig. 1:**
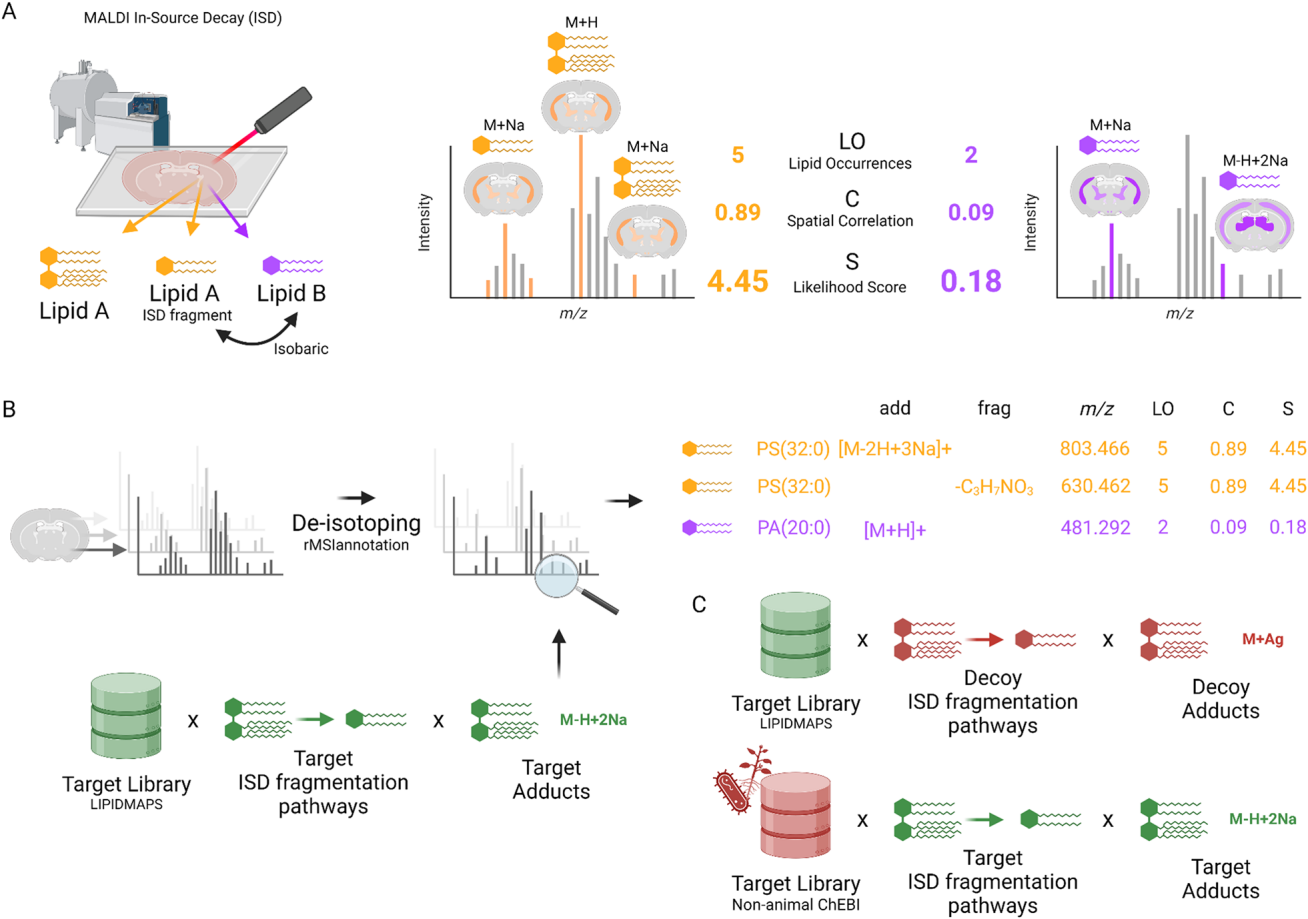
Main algorithmic foundations for the annotation of in-source fragments. **A** ISD can generate in-source fragments that overlap with endogenous lipids. The use of Lipid Occurrences (LO) and Spatial Correlation (**C**) allows rMSIfragment to rank the likelihood of isobaric lipid annotations. **B** General rMSIfragment flux diagram. **C** Two alternative decoy libraries based on highly unlikely adducts and fragmentation pathways (top) and non-animal/xenobiotic compounds (bottom)

As reported by Garate et al. [20] ISD can produce lipid fragments that overlap with endogenous lipids (Fig. 1A). rMSIfragment estimates the likelihood of each lipid by computing two metrics: lipid occurrences (LO) and spatial correlation (C). For each lipid in the library, rMSIfragment performs an exact mass search considering the adducts and in-source fragmentation pathways of the corresponding lipid class given by Garate et al. [20]. LO is the number of m/z features found. C is the mean spatial correlation (Pearson’s r) of all possible distinct pairs of m/z features found. The final ranking score is computed using the following equation:

$$S=LO\cdot(1+ C)$$

Figure 1B summarizes the complete workflow. Initially, rMSIannotation [49] is used to perform deisotoping (removal of *m/z* features considered to be isotopes). Later, all remaining *m/z* features are matched against LIPIDMAPS considering all theoretically feasible adducts and fragments. Each lipid class has a different list of theoretical adducts and in-source fragmentation pathways. The results of the annotation are stored in an R “data.frame” that can be exported to.csv for manual inspection.

During validation, rMSIfragment uses two alternative decoy libraries (Fig. 1C) to estimate the False Discovery Rate (FDR) and performance (ROC AUC) of the annotation. The first decoy library is formed by generating highly unlikely adducts and fragmentation pathways. The second decoy library replaces LIPIDMAPS with a list of compounds found in non-animal specimens and thus highly unlikely to be found in animals. Both approaches will be described more in detail in Sect. "rMSIfragment shows high performance in a target-decoy validation".

Although by default, LIPIDMAPS [40] is used to perform database searches, the user can adjust the software to use any publicly available database (e.g. HMDB, MoNA, METLIN or, NIST) or an in-house made compound database.

## Results

### rMSIfragment matches HPLC–MS annotations in human nevi samples.

As initial validation, we challenged rMSIfragment with the annotation of human nevi samples G1-G15. In a previous study [20], extracts from the same tissue were analyzed by HPLC–MS, producing a list of identified lipids in positive-ion and negative-ion polarities (Garate et al. Additional file 1: Table S2 and S3). We use these lists to estimate the performance of our automatic annotation tool.

**Fig. 2:**
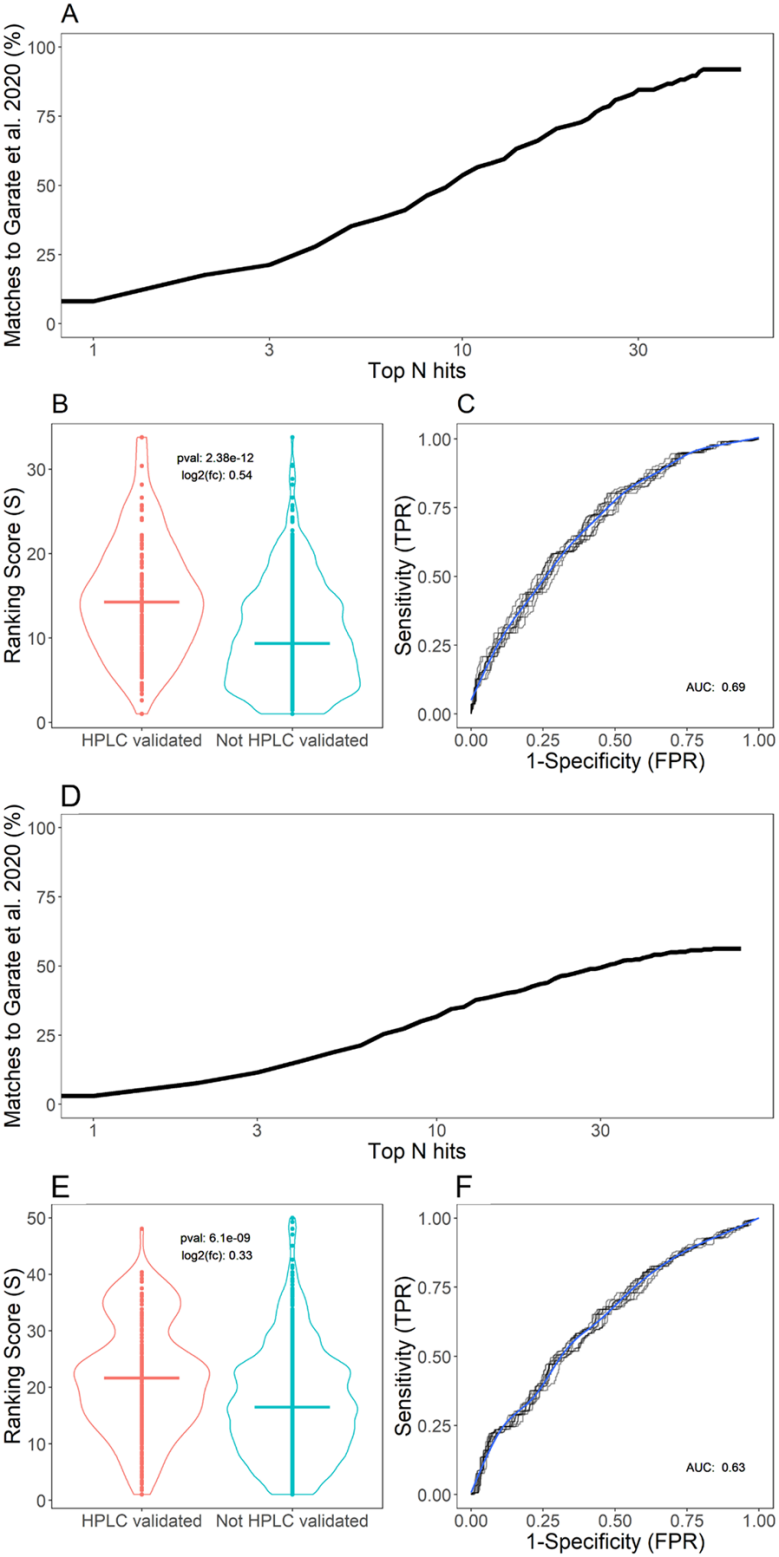
Validation of rMSIfragment against HPLC validated annotations reported by Garate et al. **A** Percentage of HPLC matches, **B** S score distribution, and **C** ROC curve for the negative polarity datasets (G9-G15). **D** Percentage of HPLC matches, **E** S score distribution, and **F** ROC curve for the positive polarity datasets (G1-G8)

Figure 2 shows the estimated performance of rMSIfragment when comparing its automatic annotations (based only on MSI data) to the list of HPLC–MS-identified lipids. To control the number of rMSIfragment annotations we propose retaining the top N annotations with the highest S score for each *m/z* feature.

In the samples acquired in negative-ion polarity (G9-G15) (Fig. 2A) rMSIfragment retrieves 91.81% of the HPLC-validated annotations reported in the original publication. To retrieve at least 50% of the HPLC annotations we need to retain the top 9 hits with the highest S score in each *m/z* feature. When only keeping the top 5 annotations rMSIfragment returns 35.92% of the HPLC-validated annotations. Figure 2B shows that the HPLC-validated annotations obtain a significantly higher S score than the annotations not validated by HPLC (p-value < 0.01, t-test). Additionally, Fig. 2C shows an ROC area under the curve (AUC) of 0.7 when using the HPLC annotations as validation.

In positive-ion polarity (G1-G8) (Fig. 2D) rMSIfragment retrieves 56.18% of all the HPLC-validated annotations. Despite the lower performance when compared to the negative-ion polarity samples, rMSIfragment is still capable of retrieving 18.56% of the annotations when focusing on the top 5 annotations for each MS feature. HPLC-validated annotations still report a significantly higher S score (p-value < 0.01, t-test) (Fig. 2E) but present a slightly worse performance at 0.63 AUC.

An alternative representation of the same results is shown in Additional file 1: Figure S2. Instead of selecting the top N annotations per *m/z* feature, we set a global likelihood score (S) threshold. A complete list of rMSIfragment annotations is provided in Additional file 2: Tables S5 and Additional file 3: Table S6.

**Fig. 3:**
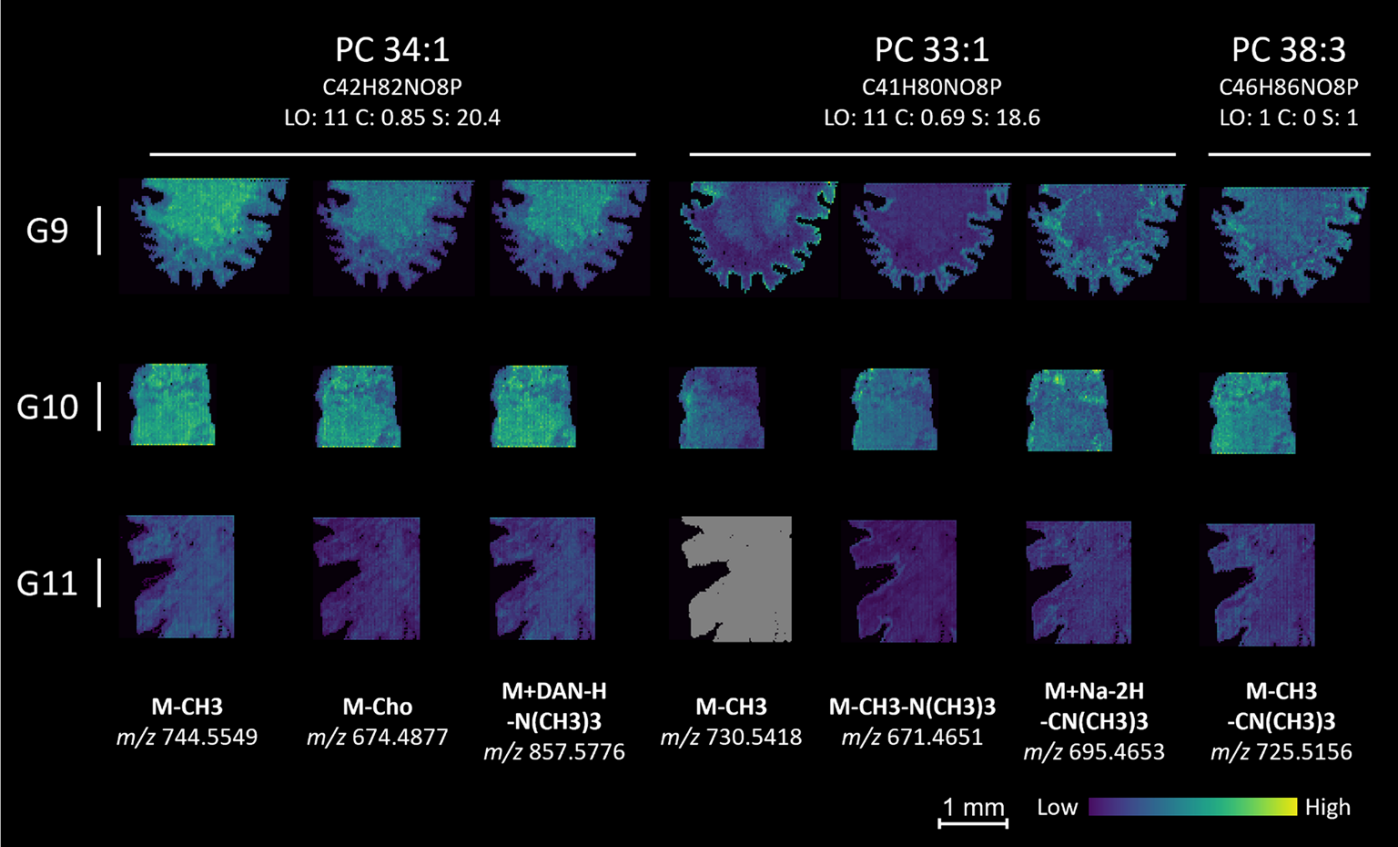
Example annotations by rMSIfragment. PC 34:1 reports a high S score and was also found by HPLC–MS in the lipid extracts. PC 33:1 shows a similarly high S score (with worse spatial correlation) but was not found by HPLC–MS. PC 38:3 was found by HPLC–MS but shows a low S score

The current validation assumes that all HPLC-validated lipids should be found in the samples and the rest should not. HPLC-MSI and MALDI-MSI are fundamentally different analytical techniques [6] and some lipids found with one technique could not be found with the other. We consider that HPLC annotations can accurately estimate the number of true positives (present and matched by rMSIfragment) and false negatives (present but not matched by rMSIfragment), but they may fail to estimate true negatives (not present and not matched by rMSIfragment) and false positives (not present but matched by rMSIfragment). Figure 3 highlights some of these differences in the annotation of three different phosphatidylcholines. PC 34:1 was found by both HPLC–MS and MALDI-MSI, PC 33:1 showed a similarly high S score but was not found by HPLC–MS, and PC 38:3 was found by HPLC–MS but shows a low S score.

Performances of 0.7 AUC might be considered modest in other fields. For instance, scores above 0.85 AUC are common in chest X-ray classification [50, 60]. However, performance depends on task complexity [51]. As rMSIfragment is the first tool addressing in-source fragmentation in MALDI-MSI, a direct benchmark against comparable tools is not possible. When compared to top MSI annotation tools like MSM [42] and METASPACE-ML [56], our 0.7 AUC performance ranks in the top quartile [56]. While not directly comparable due to their different focus, these performances offer a reliable estimate of the state of MALDI-MSI molecular annotation. Overall, rMSIfragment demonstrates confident annotation of lipid in-source fragments, attaining performances that approach the theoretical upper limit of current MSI annotation tools.

### rMSIfragment shows high performance in a target-decoy validation

In an attempt to gauge and overcome the limitations of the HPLC validation, we propose a second validation based on a target-decoy search strategy, a commonly used approach in MSI [21, 42]. This strategy runs rMSIfragment on the same MSI data using our target library (LIPIDMAPS) and a decoy library containing compounds that should not be found in the sample. The decoy library matches the size and distribution of masses of the target library, to ensure that randomly generated masses are equally likely to hit either of the two databases. The rate of matches in the decoy can then be used to estimate measures such as true positives, true negatives, false positives, false negatives, and False Discovery Rate (FDR) [17].

**Fig. 4:**
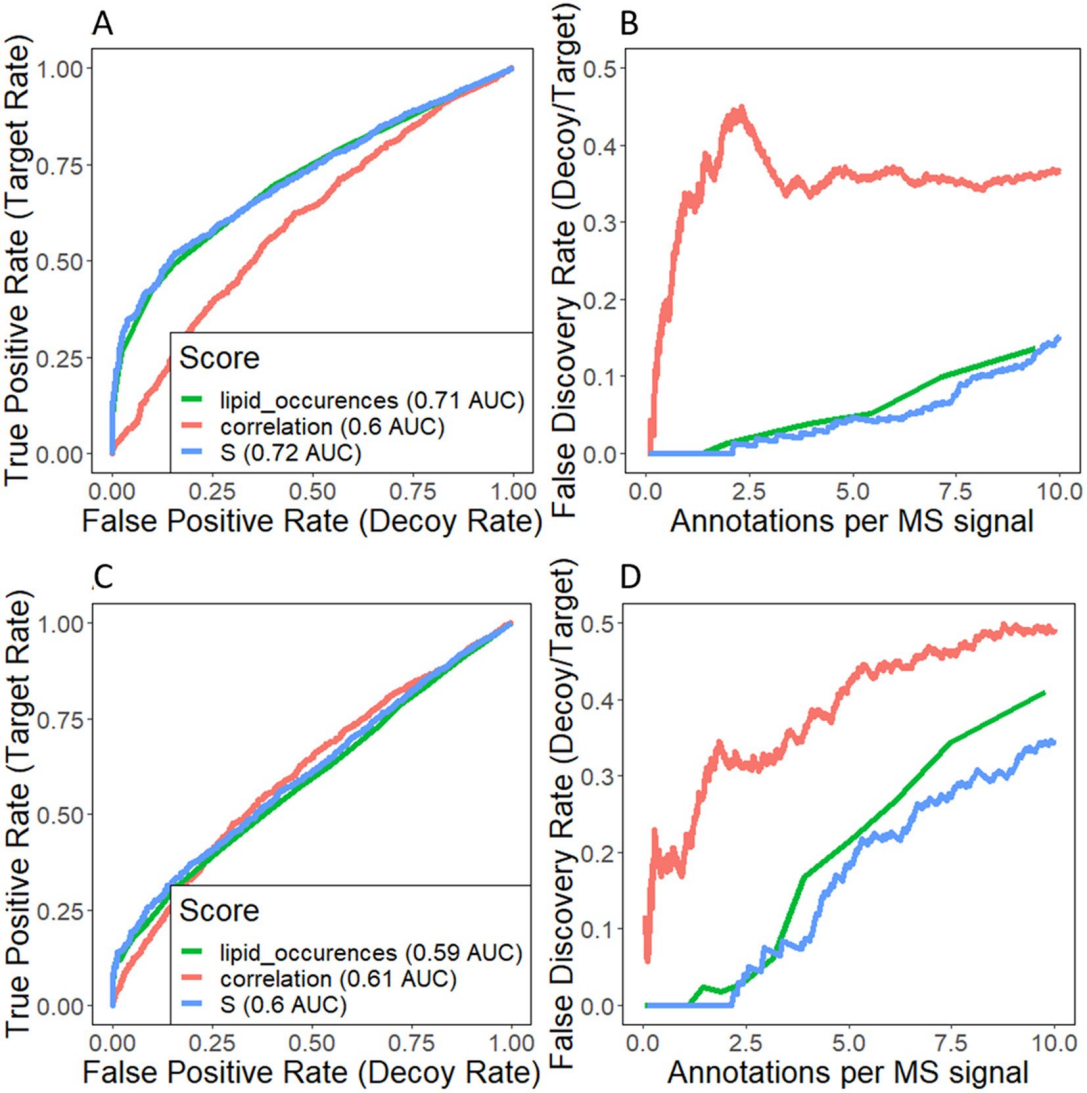
Performance estimation of the ranking scores proposed using a target-decoy validation approach. The decoy database is composed of adducts and fragmentation pathways highly unlikely to be found in the lipid classes considered. Lipid occurrences (LO): number of times a given lipid is found (including parental adducts and in-source fragments); Spatial correlation (C): The weighted mean Pearson’s correlation of all *m/z* features annotated as the same lipid. Final ranking score (S): LO * C. **A** ROC curve and **B** FDR estimation for the negative polarity datasets (G9-G15) **C** ROC curve and **D** FDR estimation for the positive polarity datasets (G1-G8)

Figure 4 shows the results of the target-decoy validation on the human nevi datasets (G1-G15) using a decoy library composed of highly unlikely adducts and fragmentation pathways, an approach adapted from pySM [42]. The classification performance obtained a value of 0.72 AUC for the negative-ion polarity datasets (Fig. 4A) and 0.6 AUC for the positive-ion polarity datasets (Fig. 4C). These results are consistent with the performances obtained in HPLC validation. When retaining the top 10 matches for each MS feature the FDR is estimated to be 14.93% in negative-ion polarity (Fig. 4B) and 34.24% in positive-ion polarity (Fig. 4D). Similarly, when only retaining the top 5 matches per MS feature the FDR is 4.5% and 17.95% respectively.

As extra validation, we created a second decoy library, a subset of ChEBI [23] containing only metabolites found in non-animal specimens (plants, algae, fungi, and bacteria) and xenobiotics. The results are summarized in Additional file 1: Figure S3. The performance on the negative-ion polarity samples is consistent with the one estimated in previous approaches (0.73 AUC). In the positive-ion polarity samples, the performance is estimated to be higher than in previous validations (0.75 AUC). In both cases, the FDR is estimated to be under 10% when retaining the top 10 matches.

The two decoy libraries provided comparable estimations of performance and FDR. Nevertheless, the definition of a decoy library based on non-animal/xenobiotic compounds (Additional file 1: Figure S3) is sample-dependent. The use of highly unlikely adducts and fragments, on the other hand, has already been discussed [42] and can be assumed to be much more sample-independent. Additionally, its performance estimates are more conservative and closely match the results obtained in the HPLC validation. For all these reasons, further validations use a decoy library composed of highly unlikely adducts and fragments.

These results further confirm that rMSIfragment can confidently annotate lipids and their fragments.

### rMSIfragment can be used under multiple experimental conditions

**Fig. 5:**
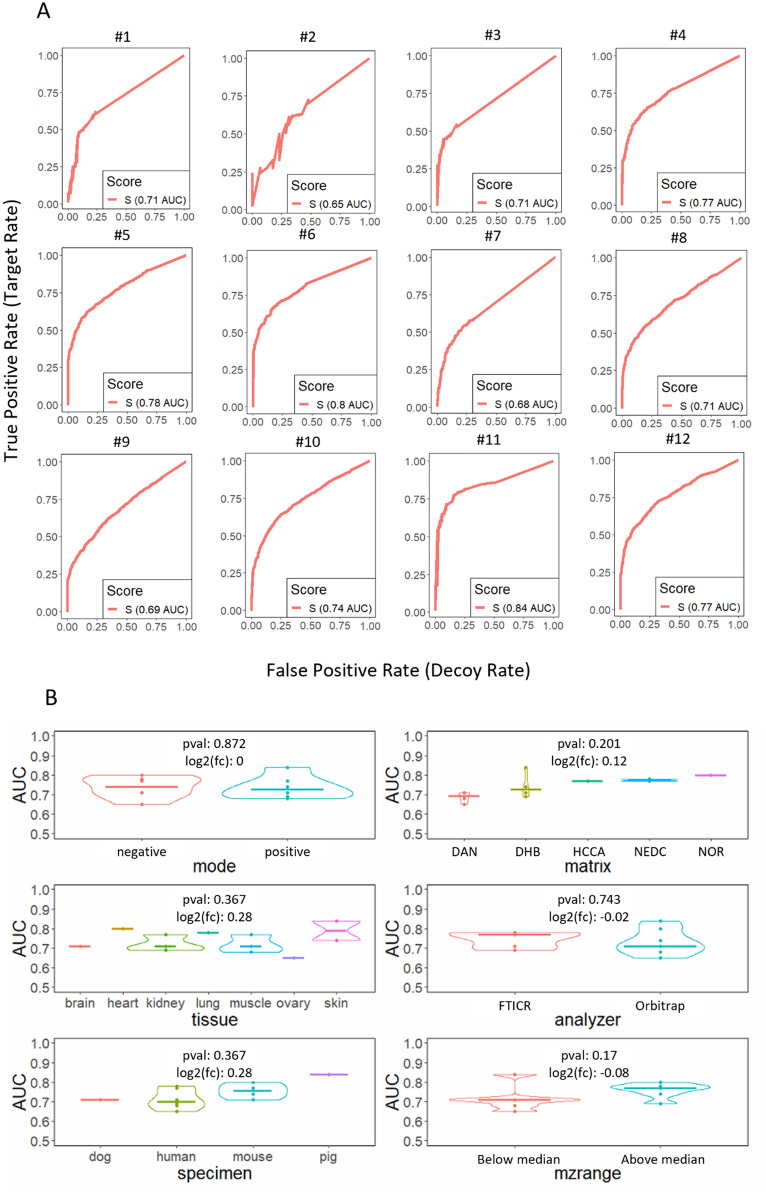
Target and decoy validation on 12 datasets publicly available on METASPACE [4]. **A** ROC curves and Area Under the Curve (AUC) for each dataset M1-M12. **B** Statistical significance tests of performance (AUC) differences across different biological (tissue and specimen), sample preparation (matrix), and instrumental (ion polarity, analyzer, *m/z* range) parameters

To determine its applicability to other experimental conditions we challenged rMSIfragment with the annotation of 12 different publicly available datasets from METASPACE (Datasets M1-M12) [4]. Since the real lipid composition of these datasets is unknown, we use the target and decoy approach based on highly unlikely adducts and fragments to estimate the performance of rMSIfragment in each dataset. Figure 5 summarizes the results. The performances ranged between 0.65 AUC and 0.84 AUC (μ = 0.74 AUC, σ = 7.5%) (Fig. 5A). These performances are comparable to the performance on human nevi samples validated with HPLC.

Additionally, when grouping the datasets according to different experimental parameters (ionization polarity, matrix, tissue, analyzer, specimen, or mass range) the differences in performance were not found to be significant (p-value > 0.1, f-test). This demonstrates that rMSIfragment applies to a wide range of experimental conditions.

### Annotation software must take into account in-source fragmentation

Major automatic annotation tools such as pySM [42], LipostarMSI [55] or rMSIannotation [49] completely overlook in-source fragmentation and almost exclusively focus on protonated and alkali ions. Ignoring in-source fragmentation during annotation could potentially lead to false annotations [8]. This is a particular concern in lipidomics where several lipids fragment in-source and become isobaric to other lipids [20].

To assess the impact of in-source fragmentation on lipid annotation we annotate datasets M1-M12 with METASPACE [4], only considering traditional MALDI adducts ([M + H]^+^, [M + Na]^+^, [M + K]^+^ in positive-ion polarity and [M-H]^−^, [M + Cl]^−^ in negative polarity). We then annotate the same datasets using rMSIfragment, considering all adducts and fragmentation pathways specified in Additional file 1: Fig. S1 and Table S1. The same version of LIPIDMAPS is used with both tools.

**Fig. 6:**
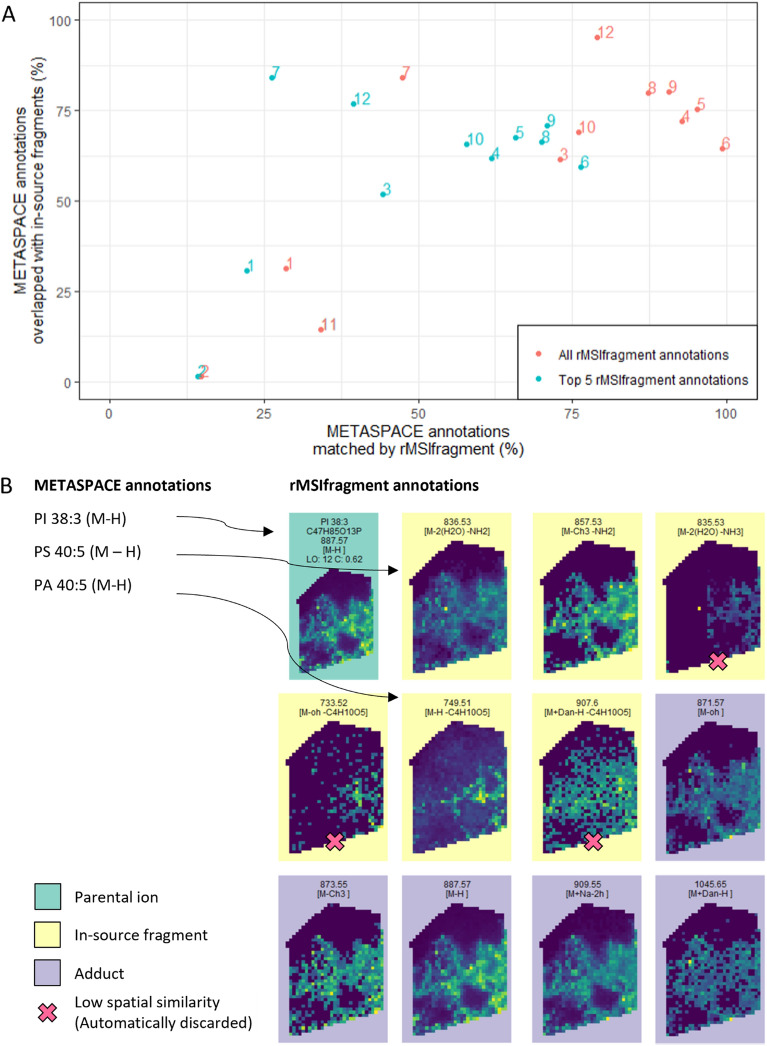
Comparison of annotation results between rMSIfragment and METASPACE **A** Bulk comparison using 12 datasets publicly available in METASPACE. The horizontal axis shows the percentage of METASPACE annotations that are matched by rMSIfragment. The vertical axis indicates the percentage of METASPACE annotations that are overlapped with at least one in-source fragment annotated by rMSIfragment. The standard FDR threshold of 0.2 was used for METASPACE annotations. rMSIfragment annotations without any threshold (red) and retaining the top 5 annotations per MS feature (blue). **B** Example comparison for a human lung biopsy (Dataset M5) where *m/z* 887.57 is annotated by both tools as PI 38:3 (M + H) (C47H85O13P). In the same dataset, rMSIfragment also finds 3 adducts and 3 in-source fragments with high spatial correlation to the parental ion. Two of these in-source fragments are overlapped with 2 METASPACE annotations ([PA 40:5 M-H]^−^, [PS 40:5 M-H]^−^)

Figure 6A summarizes the overall comparison of annotations between the 2 tools. On average, 48.6% of the annotations returned by METASPACE are also found with rMSIfragment. Interestingly, crossing the annotations also allows us to determine that, on average, 54.21% of METASPACE annotations are overlapped with at least one in-source fragment found by rMSIfragment. Additional file 1: Figure S4 shows the same results color-coded based on the different sample and experimental parameters.

To exemplify this overlap we highlight the annotation of *m/z* 887.57 from a human lung cancer biopsy prepared with NEDC and analyzed in negative polarity with an FTICR (Dataset M5) (Fig. 6B). Both tools reliably annotate this *m/z* feature as PI 38:3 (M-H)^−^. In the same dataset, rMSIfragment also finds 3 adducts ($[M-OH]$^−^, $[M-C{H}_{3}]$^−^, [$M+ Na-2H$]^−^) and 3 in-source fragments ([$M-C{H}_{3}-N{H}_{2}$]^−^, [$M-H-{C}_{4}{{H}_{10}{O}_{5}}$]^−^, [$M-2{H}_{2}O-N{{H}_{2}}$]^−^) with high spatial correlation to the parental ion. Two of these in-source fragments ([$M-H-{C}_{4}{{H}_{10}{O}_{5}}$]^−^, [$M-2{H}_{2}O-N{{H}_{2}}$]^−^) are overlapped with 2 METASPACE annotations ([PA 40:5 M-H]^−^, [PS 40:5 M-H]^−^).

These results are not a comparison between tools but rather a quantification of the negative impact that in-source fragmentation has on automatic annotation tools. Annotation tools in MSI need to take into account in-source fragmentation. Due to the fundamental limitations of MS1, software tools are unable to resolve an endogenous PA from an isobaric PA originating from the in-source fragmentation of its PS counterpart. However, rMSIfragment mitigates the issue by (1) making the user aware of the potential overlap and (2) giving a higher score to the lipid found forming other adducts and in-source fragments.

### rMSIfragment provides a molecular network to visually interpret the results

**Fig. 7:**
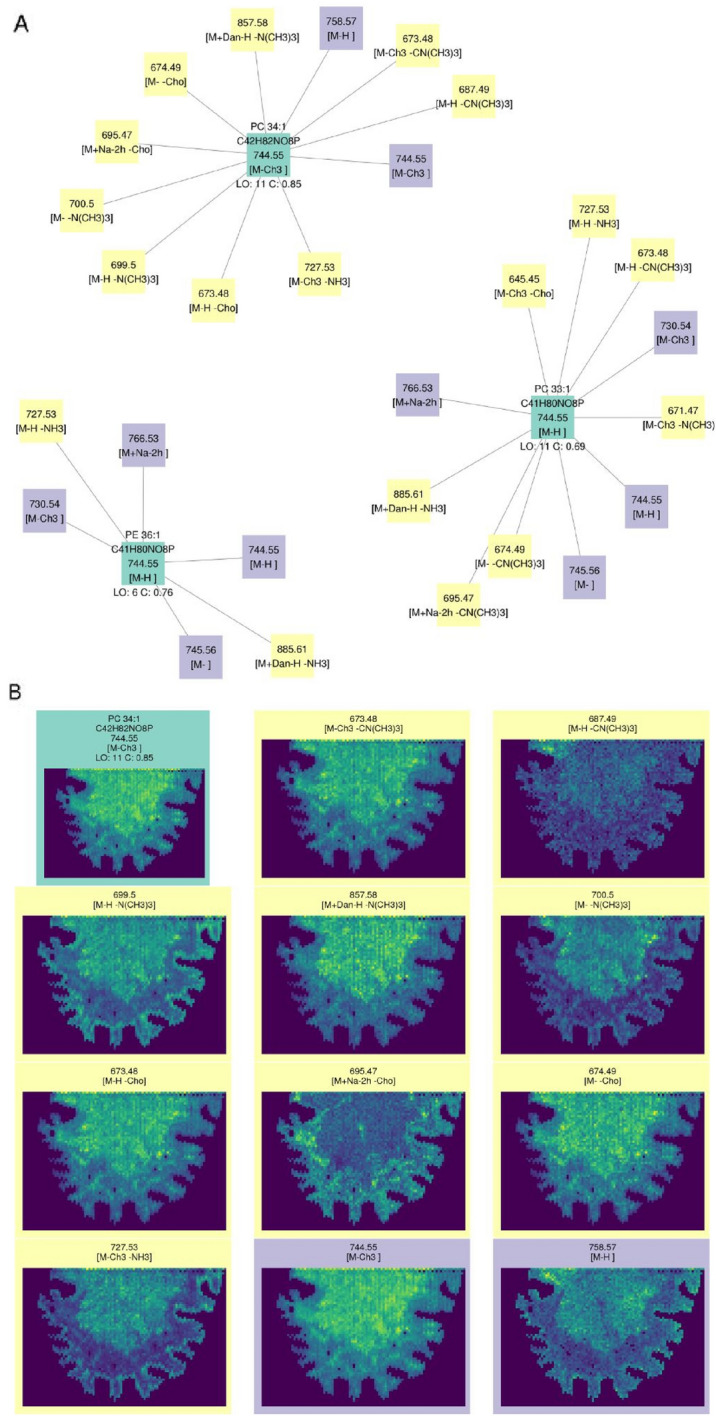
Example application and interpretation of rMSIfragment results on Dataset G9. **A** Top 3 annotations for *m/z* 744.55. Each parental annotation (green) is the center of a network including all adducts (purple) and in-source fragments (yellow) annotated in the sample. **B** Spatial representation of the top annotation (PC 34:1 [M-CH3])

Figure 7 shows an example exploration of the annotation results using the rMSIfragment GUI. The top 3 annotations for *m/z* 744.55 are shown as individual molecular networks including adducts (purple) and in-source fragments (yellow) (Fig. 7A). The number of lipid occurrences (LO) and spatial correlation (C) are shown as the main metrics to filter out unlikely lipids. When selecting the desired molecular network the user can view the spatial distribution of all adducts and in-source fragments.

## Discussion and conclusions

We have demonstrated the performance of rMSIfragment on 15 human nevi datasets with two orthogonal approaches: (1) matching its annotations to HPLC and (2) using a target-decoy approach. Both approaches yield similar performance estimates (0.7 AUC and 0.6 AUC for the samples acquired in negative and positive polarity). rMSIfragment is the first tool addressing in-source fragmentation and cannot be directly benchmarked. However, our results rank in the upper quartile of performances reported by leading tools in metabolite annotation [42, 56], a closely related task.

We also annotated 12 publicly available datasets covering a wide combination of samples and experimental setups. The performances obtained are comparable and often better than the ones obtained on the human nevi datasets. Additionally, rMSIfragment shows a high lipidome coverage overlap comparable to available annotation tools like METASPACE [4]. These findings suggest that rMSIfragment can consistently annotate lipids and their in-source fragments under different experimental conditions.

One key highlight of our study is the importance of considering in-source fragmentation pathways when performing molecular annotation. We have found that overlooking ISD pathways can lead to up to 75% of the reported lipid annotations to be overlapped with at least one in-source fragment. rMSIfragment mitigates this issue through two mechanisms: (1) unlikely lipids with low occurrences (number of adducts and in-source fragments) and poor spatial correlation are filtered out, and (2) the user is aware of the overlap, allowing them to be cautious with their interpretation of the automated annotations.

We propose three avenues to increase the performance of the future generation of MALDI-MSI in-source fragmentation annotation tools: (1) leveraging known ion suppression effects between different lipid classes, (2) compiling MALDI-ISD or MALDI-MS/MS libraries and, (3) deploying Machine Learning (ML) and Deep Learning (DL) models.

Ion suppression effects strongly favor certain classes of lipids, difficulting the analysis of suppressed species [11]. In positive polarity, PC species display stronger signals than other lipids (PE, PS, PG, or PI). In negative polarity, the effect is reversed and PC species show lower signals than other lipids. These interactions have been characterized in the past [11] and could be leveraged to define a new ranking score to filter out unlikely lipid annotations.

Previous LC–MS studies [61] induce increased ESI in-source fragmentation to yield fragmentation patterns similar to those present in MS/MS libraries like METLIN [52] to aid in molecular identification. In a preliminary exploration, we concluded that public MS/MS libraries were not directly applicable to MALDI-MSI for two reasons: (1) MALDI and ESI are not always directly comparable, and (2) the fragmentation of common MALDI adducts such as [M + Na] + and [M + K] + are underrepresented in MS/MS libraries (< 10%). The compilation of MALDI-ISD libraries could overcome these limitations. This community-wide effort would help better characterize MALDI-ISD in a wide range of biomolecules. Alternatively, a MALDI-MS/MS library, which may be a more urgent interest of the MALDI community, could already provide enough information. These two libraries would be invaluable tools to foster the development of the next generation of ISD annotation algorithms and models in MALDI-MSI.

Finally, in spite of their success in MALDI-MSI tasks like tumor classification [9], clustering [62], and image registration [44], ML and DL models have yet to be consistently deployed for molecular annotation [3, 8]. The performance of rMSIfragment could potentially be improved l,leveraging ML to suggest optimal functions of our predefined metrics (LO and C to compute the final score (S, following the strategy proposed by METASPACE-ML [56]. Taking a step further, new metrics could be defined to better capture spectral and spatial similarities of in-source fragments. However, DL has only attained modest improvements in MS image colocalization [41]. From our perspective, creating DL models for MALDI in-source fragmentation holds the greatest promise for enhancing annotation performance, building upon the achievements of in-silico MS/MS fragmentation tools like Metfrag [47], CFM-ID (F. [57, 59], and Sirius [16].

In conclusion, neglecting in-source fragmentation leads to an increased number of false lipid annotations. rMSIfragment mitigates this effect by prioritizing annotations of lipids found forming multiple adducts and in-source fragments.

## Materials and methods

A total of 27 different datasets were used to validate this study. Human nevi samples acquired in positive (G1-G8) and negative (G9-G15) ion polarity from a previous study [20] were used to perform HPLC validation and determine the best target-decoy strategy for further validation. Publically available METASPACE [4] datasets M1-M12 were used to demonstrate the applicability of rMSIfragment to different sample types and experimental conditions. Additional file 1: Table S4 summarizes the main processing parameters for each of the 27 datasets.

### Sample preparation

Human nevi tissue sections G1-G15 were already used in a previous study [20]. The Euskadi Ethics Committee approved the study protocol and it conformed to the Helsinki Declaration. The biopsies were embedded in OCT and sectioned at 16 μm thickness. The sections were covered with MALDI matrices 2-Mercaptobenzothiazole (MBT) and 1,5-Diaminonaphthalene (DAN) for positive and negative-ion modes respectively. Both matrices were sublimated onto the sample using an Ace Glass 8023 Glass Sublimator.

### MALDI‑MSI acquisition

Mass spectra were acquired using an LTQ-Orbitrap XL mass spectrometer (ThermoFisher, MA, USA), equipped with a custom MALDI source with an N2 laser [20]. Low laser energy was used to prevent excessive fragmentation. The spectrum at each pixel is the result of averaging two micro-scans of 10 shots. Positive-ion polarity experiments had an *m/z* range of 480 − 1100 Da while negative-ion polarity experiments had a range of 550 − 1200 Da. The mass resolving power was set to 30 000 at *m/z* 400. The step size was 25 μm.

### MSI data processing

Datasets G1-G15, originally in.RAW format (Thermo Fischer), were exported to.mzML using ProteoWizard msConvert [1], and later converted to.imzML [48] using imzMLConverter [43]. The software rMSIproc [45] was used to process the data and generate a peak matrix in centroid mode. The default processing parameters were used. The Signal-to-Noise Ratio (SNR) threshold was set to 5 and the Savitzky– Golay smoothing had a kernel size of 7. Peaks appearing in less than 5% of the pixels were filtered out. Peaks within a window of 6 data points or scans were binned together as the same mass peak.

Datasets M1-M12, already in centroid mode.imZML, were imported using rMSIproc [45].

Deisotoping was performed using rMSIannotation [49]. No data normalization was performed. Data were visualized and explored using rMSI [46].

Statistical significance is tested using a t-test or an f-test for the comparison of 2 and 3 + groups respectively.

### Supplementary Information

**Additional file 1: Figure S1.** Lipid fragmentation pathways. Reproduced with the permission of Garate et al. 2020. **Figure S2.** Automatic annotation with rMSIfragment validated with HPLC in human nevi samples (Garate et al. 2020). Percentage of HPLC validated matches (Garate et al. 2020) against increasing ranking score (S) threshold (blue). **(A)** Samples G9-G15 (negative-ion polarity). **(B)** Samples G1-G8 (positive-ion polarity).** Figure S3.** Performance estimation of the Ranking scores proposed using a Target Decoy Validation approach. The Decoy database is composed of metabolites and lipids unlikely to be found in non-animal specimens (plants, algae, fungi, and bacteria) and xenobiotics. (A) ROC and (B) FDR estimation on samples G9-G15 (negative-ion polarity). (C) ROC and (D) FDR estimation on samples G1-G8 (positive-ion polarity). **Figure S4. **METASPACE annotations overlapped with in-source fragments vs METASPACE annotations matched by rMSIfragment color-coded based on: **(A)** Ion polarity **(B)** MALDI matrix **(C)** Tissue type **(D)** Analyzer **(E)** Mean *m/z*. **Table S1.** Lipid adduct formation in positive-ion polarity. Reproduced with the permission of Garate et al. 2020. **Table S2.** Lipid adduct formation in negative-ion polarity. Reproduced with the permission of Garate et al. 2020. **Table S3.** Example rMSIfragment output. **Table S4.** List of the 27 MALDI MSI datasets used for validation. Sample type, sample preparation, and MALDI-MSI acquisition parameters.

**Additional file 2: Table S5: **Complete list of annotations using rMSIfragment (Samples G1-G8).

**Additional file 3: Table S6: **Complete list of annotations using rMSIfragment (Samples G9-G15).

## Data Availability

The platform-independent R package rMSIfragment presented in this publication is freely available under the terms of the GNU General Public License v3.0 at https://github.com/gbaquer/rMSIfragment. The datasets supporting the conclusions of this article are available in the Mendeley Data repository: https://doi.org/10.17632/53grw3ys6y.1 (Datasets G1—G15). Datasets M1-M12 are available at https://metaspace2020.eu/ (References provided in Additional file 1: Table S1). rMSIfragment is available under the GPLv3 at www.github.com/gbaquer/rMSIfragment.
