# Supplementary material for: rMSIfragment: improving MALDI-MSI lipidomics through automated in-source fragment annotation
Source: J Cheminform. 2023 Sep 15;15:80. doi: 10.1186/s13321-023-00756-2 (PMC10504721; doi:10.1186/s13321-023-00756-2)
Supplement: Supplementary file 1 — Additional file 1: Figure S1. Lipid fragmentation pathways. Reproduced with the permission of Garate et al. 2020. Figure S2. Automatic annotation with rMSIfragment validated with HPLC in human nevi samples (Garate et al. 2020). Percentage of HPLC validated matches (Garate et al. 2020) against increasing ranking score (S) threshold (blue). (A) Samples G9-G15 (negative-ion polarity). (B) Samples G1-G8 (positive-ion polarity). Figure S3. Performance estimation of the Ranking scores proposed using a Target Decoy Validation approach. The Decoy database is composed of metabolites and lipids unlikely to be found in non-animal specimens (plants, algae, fungi, and bacteria) and xenobiotics. (A) ROC and (B) FDR estimation on samples G9-G15 (negative-ion polarity). (C) ROC and (D) FDR estimation on samples G1-G8 (positive-ion polarity). Figure S4. METASPACE annotations overlapped with in-source fragments vs METASPACE annotations matched by rMSIfragment color-coded based on: (A) Ion polarity (B) MALDI matrix (C) Tissue type (D) Analyzer (E) Mean m/z. Table S1. Lipid adduct formation in positive-ion polarity. Reproduced with the permission of Garate et al. 2020. Table S2. Lipid adduct formation in negative-ion polarity. Reproduced with the permission of Garate et al. 2020. Table S3. Example rMSIfragment output. Table S4. List of the 27 MALDI MSI datasets used for validation. Sample type, sample preparation, and MALDI-MSI acquisition parameters. [file 13321_2023_756_MOESM1_ESM.docx]

Additional information

# 1. Additional figures


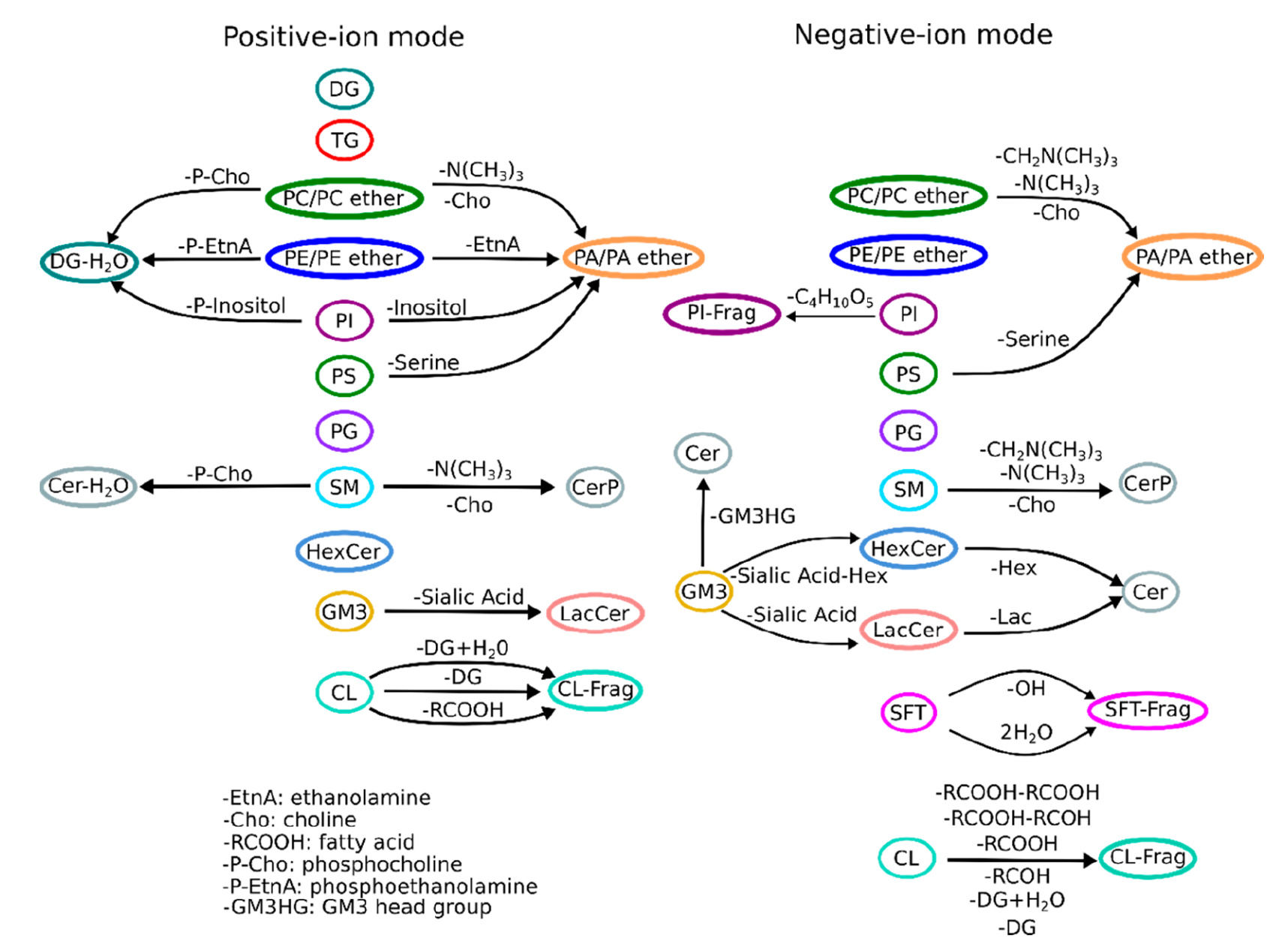


**Figure S1.** Lipid fragmentation pathways. Reproduced with the permission of Garate et al. 2020.


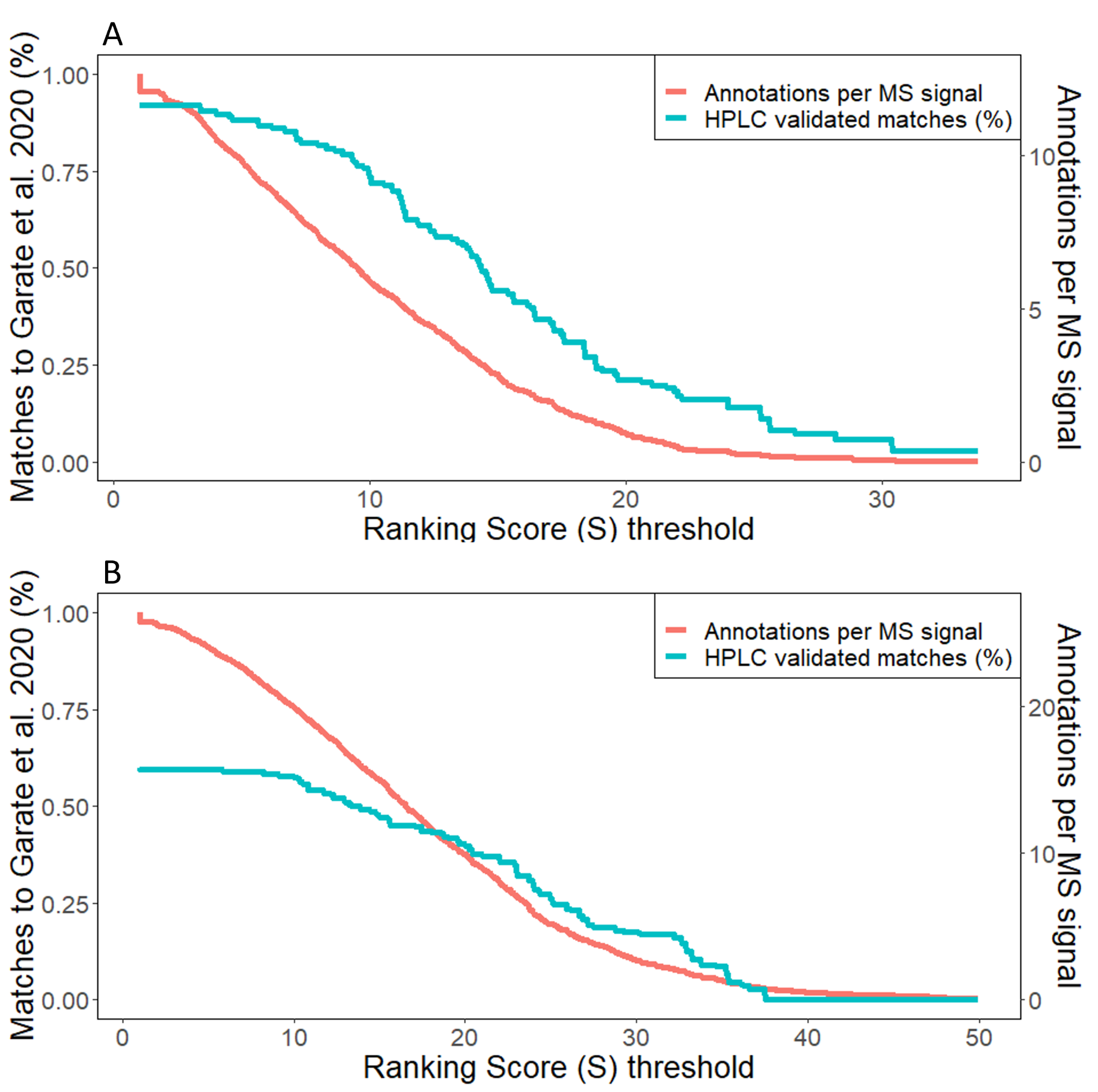


**Figure S2.** Automatic annotation with rMSIfragment validated with HPLC in human nevi samples (Garate et al. 2020). Percentage of HPLC validated matches (Garate et al. 2020) against increasing ranking score (S) threshold (blue). **(A)** Samples G9-G15 (negative-ion polarity). **(B)** Samples G1-G8 (positive-ion polarity).

**
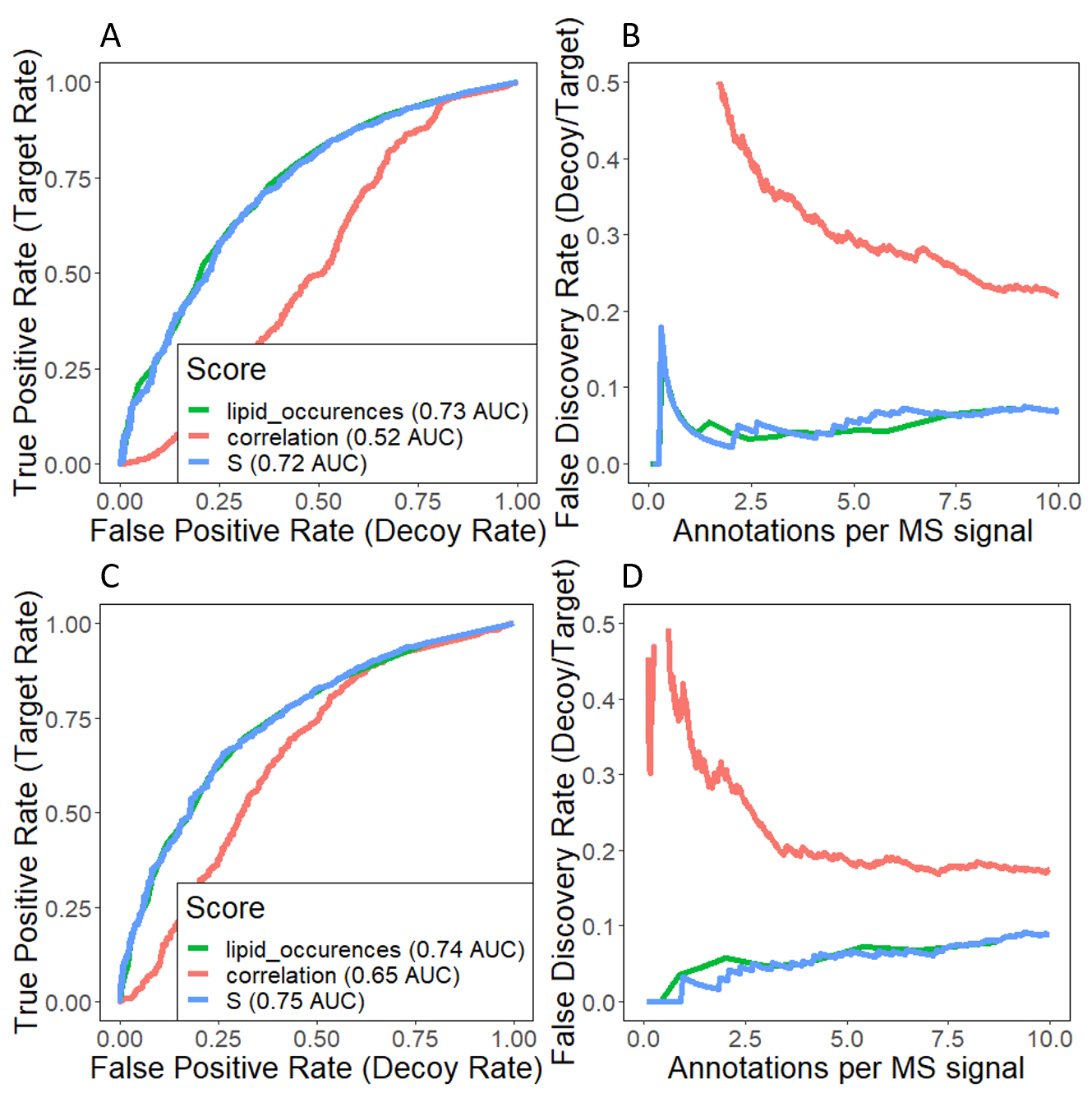
 Figure S3.** Performance estimation of the Ranking scores proposed using a Target Decoy Validation approach. The Decoy database is composed of metabolites and lipids unlikely to be found in non-animal specimens (plants, algae, fungi, and bacteria) and xenobiotics. (A) ROC and (B) FDR estimation on samples G9-G15 (negative-ion polarity). (C) ROC and (D) FDR estimation on samples G1-G8 (positive-ion polarity).


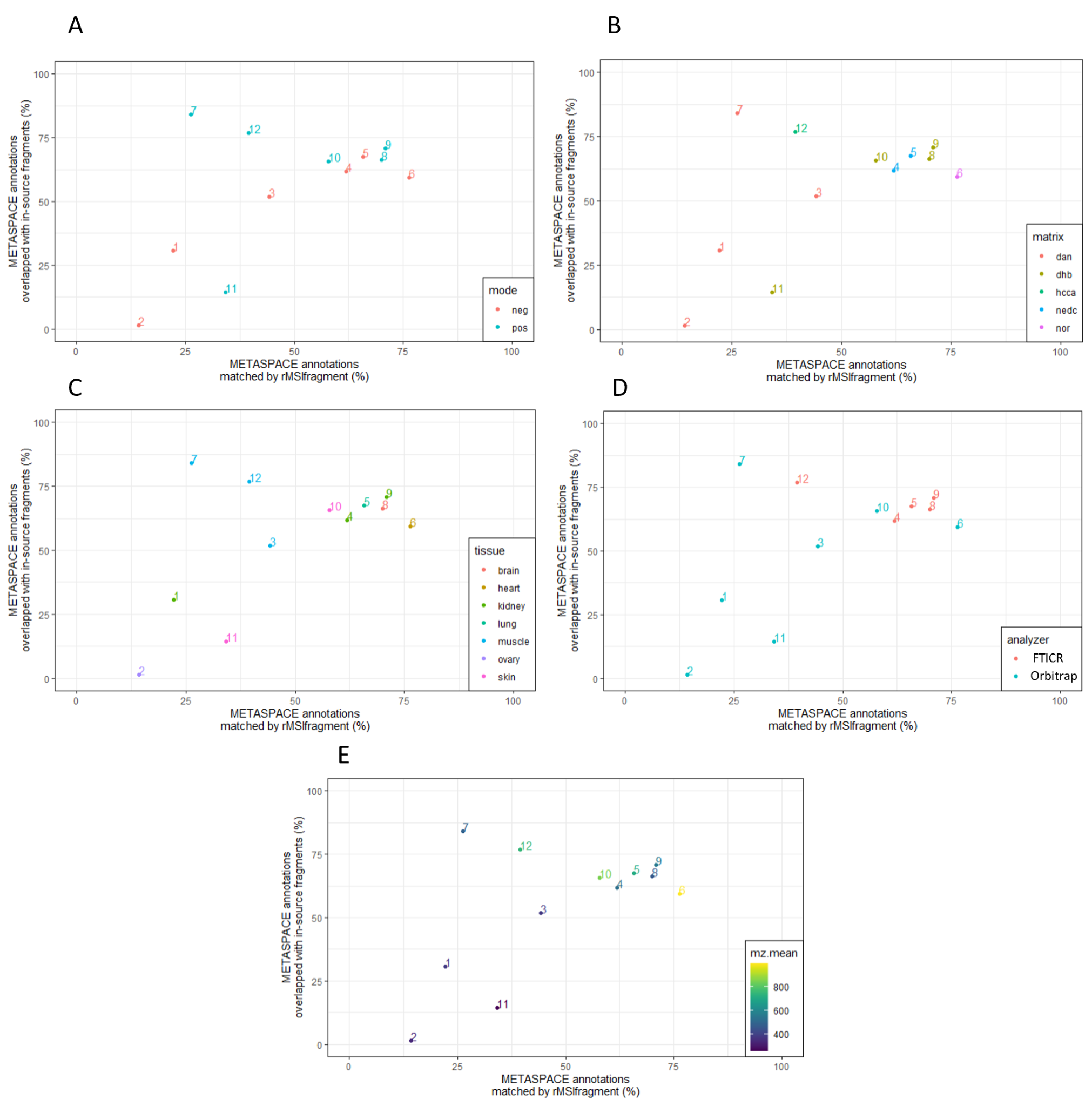


**Figure S4.** METASPACE annotations overlapped with in-source fragments vs METASPACE annotations matched by rMSIfragment color-coded based on: **(A)** Ion polarity **(B)** MALDI matrix **(C)** Tissue type **(D)** Analyzer **(E)** Mean *m/z*

# 2. Additional tables

**Table S1.** Lipid adduct formation in positive-ion polarity. Reproduced with the permission of Garate et al. 2020

**
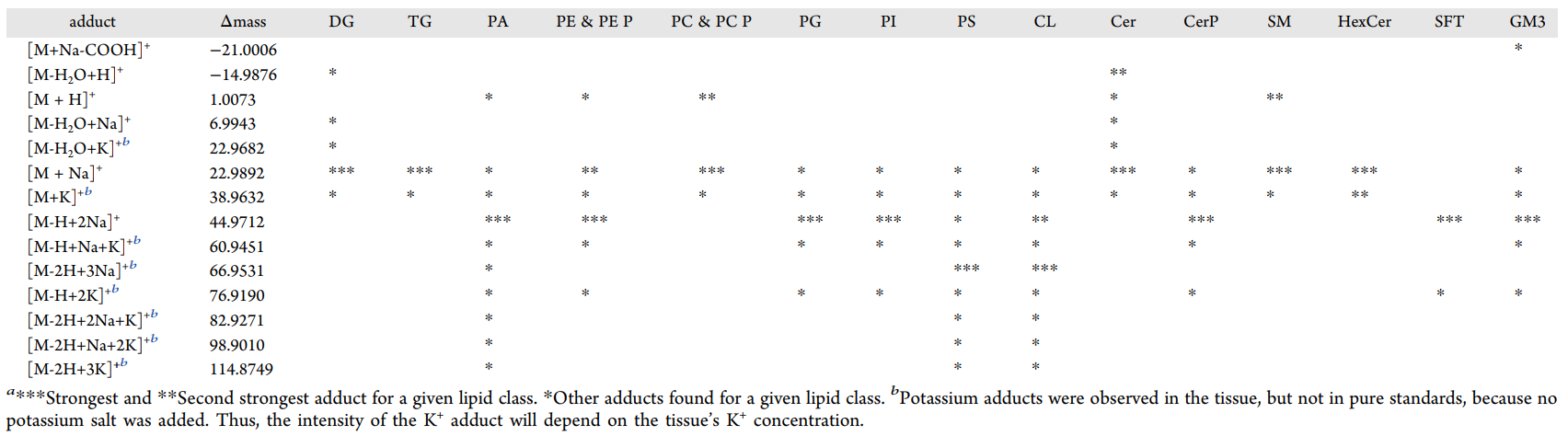
**

**Table S2.** Lipid adduct formation in negative-ion polarity. Reproduced with the permission of Garate et al. 2020


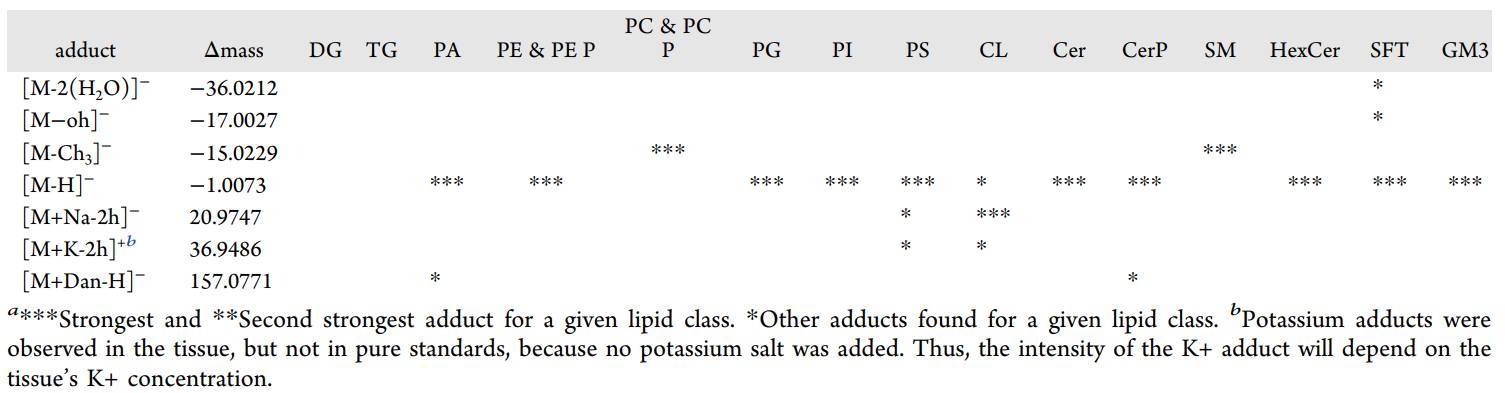


**Table S3.** Example rMSIfragment output


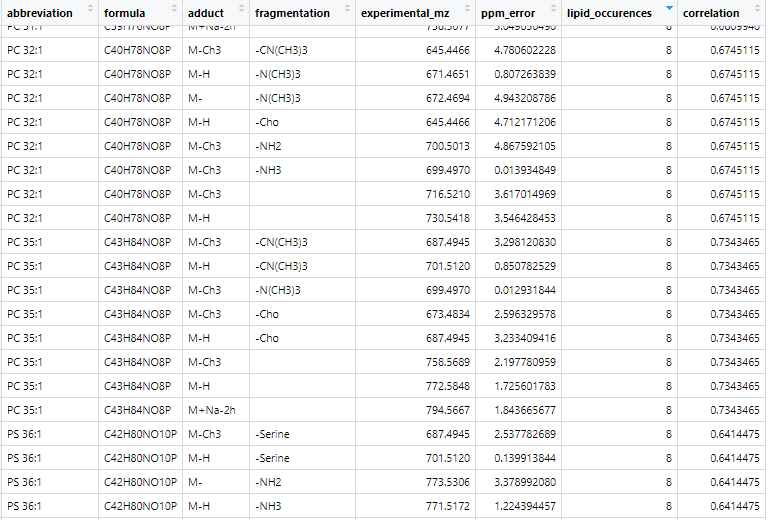


**Table S4.** List of the 27 MALDI MSI datasets used for validation. Sample type, sample preparation, and MALDI-MSI acquisition parameters.

| **No.** | **Species** | **Tissue type** | **Matrix deposition** | **Lateral Res. (um)** | ***m/z* range** | **Mass spectrometer** | **Acq. Mode** | **Notes** | **Ref.** |
| --- | --- | --- | --- | --- | --- | --- | --- | --- | --- |
| G1-G8 | *Homo sapiens sapiens* | Nevus | MBT, Ace Glass 8023 Glass Sublimator, 10 min | 25 | 480-1100 | ThermoFisher™ LTQ-Orbitrap XL | Positive / Profile | 7 replicates | [(Garate et al. 2020)](https://paperpile.com/c/VDGkG1/ab9O) |
| G9-G15 | *Homo sapiens sapiens* | Nevus | DAN, Ace Glass 8023 Glass Sublimator, 10 min | 25 | 550−1200 | ThermoFisher™ LTQ-Orbitrap XL | Negative / Profile | 7 replicates | [(Garate et al. 2020)](https://paperpile.com/c/VDGkG1/ab9O) |
| M1 | *Canis familiaris* | Kidney | DAN, TM sprayer | Not Specified | 200 - 915 | Orbitrap | Negative / Centroid |  | [(Alexandrov et al. 2019)](https://paperpile.com/c/VDGkG1/smUH) |
| M2 | *Homo sapiens sapiens* | Ovary | DAN, TM sprayer | Not Specified | 200 - 645 | Orbitrap | Negative / Centroid |  | [(Alexandrov et al. 2019)](https://paperpile.com/c/VDGkG1/smUH) |
| M3 | *Mus musculus* | Muscle | DAN, TM sprayer | Not Specified | 200 - 890 | Orbitrap | Negative / Centroid |  | [(Alexandrov et al. 2019)](https://paperpile.com/c/VDGkG1/smUH) |
| M4 | *Homo sapiens sapiens* | Kidney | NEDC, TM sprayer | 50 | 300 - 1500 | FTICR | Negative / Centroid |  | [(Alexandrov et al. 2019)](https://paperpile.com/c/VDGkG1/smUH) |
| M5 | *Homo sapiens sapiens* | Lung | NEDC, HTX M5 sprayer | 40 | 400 - 1500 | FTICR | Negative / Centroid |  | [(Alexandrov et al. 2019)](https://paperpile.com/c/VDGkG1/smUH) |
| M6 | *Mus musculus* | Heart | Norharmane, HTX TM sprayer | 30 | 400 - 1945 | Orbitrap | Negative / Centroid |  | [(Alexandrov et al. 2019)](https://paperpile.com/c/VDGkG1/smUH) |
| M7 | *Homo sapiens sapiens & Mus musculus* | Cervix & Muscle Coculture | DAN, TM sprayer | Not Specified | 200 - 1080 | Orbitrap | Positive / Centroid |  | [(Alexandrov et al. 2019)](https://paperpile.com/c/VDGkG1/smUH) |
| M8 | *Homo sapiens sapiens* | Brain | DHA, Spray robot | Not Specified | 100 - 1465 | FTICR | Positive / Centroid |  | [(Alexandrov et al. 2019)](https://paperpile.com/c/VDGkG1/smUH) |
| M9 | *Homo sapiens sapiens* | Kidney | DHB, TM sprayer | 50 | 250 - 1300 | FTICR | Positive / Centroid |  | [(Alexandrov et al. 2019)](https://paperpile.com/c/VDGkG1/smUH) |
| M10 | *Mus musculus* | Skin | DHB, Airbrush | 60 | 400 - 1600 | Orbitrap | Positive / Centroid |  | [(Alexandrov et al. 2019)](https://paperpile.com/c/VDGkG1/smUH) |
| M11 | *Sus domesticus* | Skin | DHB, Airbrush | 30 | 70 - 400 | Orbitrap | Positive / Centroid |  | [(Alexandrov et al. 2019)](https://paperpile.com/c/VDGkG1/smUH) |
| M12 | *Mus musculus* | Left upper arm | CHCA, HTX | 50 | 155 - 1970 | FTICR | Positive / Centroid |  | [(Alexandrov et al. 2019)](https://paperpile.com/c/VDGkG1/smUH) |
